# Phenotypic switching of vascular smooth muscle cells in the ‘normal region’ of aorta from atherosclerosis patients is regulated by *miR‐145*

**DOI:** 10.1111/jcmm.12825

**Published:** 2016-03-15

**Authors:** Yu‐nan Zhang, Bao‐dong Xie, Lu Sun, Wei Chen, Shu‐Lin Jiang, Wei Liu, Fei Bian, Hai Tian, Ren‐Ke Li

**Affiliations:** ^1^ Department of Cardiovascular SurgerySecond Affiliated Hospital of Harbin Medical UniversityHarbinChina; ^2^ Key Laboratories of Myocardial Ischemia Mechanism and TreatmentHarbin Medical UniversityMinistry of EducationHarbinChina; ^3^ Toronto General Research InstituteUniversity Health Network and Department of SurgeryDivision of Cardiac SurgeryUniversity of TorontoTorontoONCanada

**Keywords:** *miR‐145*, VSMCs, atherosclerosis, proliferation, phenotypic switch

## Abstract

Switching of vascular smooth muscle cells (VSMCs) from a contractile phenotype to an adverse proliferative phenotype is a hallmark of atherosclerosis or vascular restenosis. However, the genetic modulators responsible for this switch have not been fully elucidated in humans nor have they been correlated with clinical abnormalities. This study investigated genetic mechanisms involved in phenotypic switching of VSMCs at non‐defect areas of the aorta in patients with atherosclerosis. Aortic wall samples were obtained from patients with (*N* = 53) and without (*N* = 27) atherosclerosis undergoing cardiovascular surgery. Vascular smooth muscle cell cultures were generated, and expression of *microRNA‐145* (*miR‐145*), its target gene Kruppel‐Like Factor 5 (KLF5) and Myocardin (MYOCD, a smooth muscle‐specific transcriptional coactivator) were analysed using RT‐qPCR, along with expression of relevant proteins. Vascular smooth muscle cells were transduced with *miR‐145* inhibitor and mimic to determine the effect of *miR‐145* expression on VSMC proliferation. *miR‐145* expression decreased while KLF5 expression increased in atherosclerotic aortas. Atherosclerotic samples and VSMCs had decreased expression of contractile markers calponin and alpha smooth muscle actin (α‐SMA) and MYOCD. *miR‐145* inhibitor‐transduced VSMCs from non‐atherosclerotic patients showed decreased expression of calponin and α‐SMA and increased proliferation compared with non‐transduced controls, and these levels were close to those of atherosclerotic patients. *miR‐145* mimic‐transduced VSMCs from atherosclerotic patients showed increased expression of calponin and α‐SMA and decreased proliferation compared with non‐transduced controls, and these levels were close to those found in non‐atherosclerotic patients. These data demonstrate that *miR‐145* modulates the phenotypic switch of VSMCs from a contractile to a proliferative state *via* KLF5 and MYOCD in atherosclerosis.

## Introduction

Atherosclerosis is one of the most common cardiovascular diseases and is associated with high morbidity and mortality 1. Inflammation, vascular smooth muscle cell (VSMC) phenotypic modulation and atheroma plaque formation are the main pathological characteristics of this condition. Deregulation of VSMC function plays a critical role in the pathogenesis of many proliferative vascular diseases, including hypertension 2 and atherosclerosis 1. Unlike some terminally differentiated cells, VSMCs maintain remarkable phenotypic plasticity. Within mature blood vessels, VSMCs rarely proliferate or migrate. Rather, they mainly perform contractile functions and thus express a variety of smooth muscle cell (SMC)‐specific contractile markers, including α‐SMA and calponin. However, in response to injury, VSMCs undergo phenotypic modulation, characterized by decreased contractile marker expression and increased proliferation, migration and extracellular matrix synthesis 3. Control of VSMC differentiation/maturation and regulation of its responses to stimulators is involved in many factors and signalling pathways that have not been fully elucidated 4. Therefore, understanding VSMC phenotypic modulation may reveal novel therapeutic targets for the treatment of vascular diseases.

microRNAs (miRNAs) were recently reported to be involved in the process of VSMC phenotype switching. microRNAs are single‐stranded, non‐coding RNAs, 22 nucleotides in length, which typically function to induce degradation or translational repression of specific target mRNAs 5, 6, 7. Roles for these endogenous fine‐tuning regulators, most of which are inhibitors, have been primarily described in cancer, but recent reports demonstrated a direct role for miRNAs in the development of vascular pathologies, including atherosclerosis 8, 9. microRNA expression is abundant in vascular walls and miRNAs participate in various VSMC functional pathways 10. For example, *miR‐155*,* miR‐21* and *miR‐126* have been shown to contribute to vascular inflammation 11. *miR‐145* is abundantly expressed in the vessel wall and can modulate vascular neointimal lesion formation in rats 12, 13. Recently, it has been shown that *miR‐145* is dysregulated in mouse models of pulmonary arterial hypertension and that its upregulation plays an important role in vessel remodelling 14. Specifically, *miR‐145* limits intimal thickening by decreasing neointimal formation in response to angioplasty type vascular injury through promoting VSMC phenotype switching from synthetic to contractile 15. One of the most striking observations revealed by Cordes *et al*. was that *miR‐143/145* cooperatively target a network of transcriptions factors such as KLF5, KLF4 and MYOCD to promote differentiation and repress proliferation of VSMCs 16.

The role of *miR‐145* in cardiovascular pathophysiology and atherosclerosis development in humans has been explored 17. It has been shown that there are profound differences in the expression of 5 miRNAs including *miR‐145* (*miRNA‐100*,* miRNA‐127*,* miRNA‐133a* and *miRNA‐133b*) in symptomatic *versus* asymptomatic plaques. In a subsequent study, the same group found that *miR‐145* was significantly more expressed in the atherosclerotic plaques of hypertensive patients than control plaques 2. However, the expression of *miR‐145* in human aortic walls (‘normal region’) from patients with atherosclerosis and its role in the phenotypic switching of VSMCs have not been fully elucidated. In this study, we directly compared the expression levels of *miR‐145* along with the downstream mediators/contractile proteins in ‘normal’ aortic wall samples from atherosclerotic and the non‐atherosclerotic patients. The effects of *miR‐145* inhibition or promotion on VSMC phenotypic switch were examined.

## Materials and methods

### Tissue collection

Eighty patients diagnosed with coronary heart disease (CR, *n* = 42), aortic dissection disease (AR, *n* = 11), congenital heart disease (CN, *n* = 9) or valvular heart disease (VL, *n* = 18) underwent surgery at the Second Affiliated Hospital of Harbin Medical University from January 2012 to September 2014. The use of all human tissue samples was approved by the ethical committee at the Second Affiliated Hospital of Harbin Medical University, and donors gave their written informed consent. These investigations were conducted according to the principles of the Declaration of Helsinki. During surgery, tissue samples were collected from the anterior wall of the ascending aorta, ~2 cm above the aortic ring.

### VSMC culture

Tissue samples were rinsed 3–4 times in Hank's balanced salt solution at 4°C to remove blood clots. The endothelium and adventitia were gently stripped and the tunica media cut into 1 mm^2^ explants that were digested in a thermostatic oscillator at 37°C for 1 hr with 0.25% collagenase type II (Gibco, Big Cabin, OK, USA) and 0.5% elastase type II (Gibco). Following digestion, the explants were inoculated into culture bottles with 1.5–2 ml DMEM (Hyclone, Novato, CA, USA) supplemented with 10% (v/v) foetal bovine serum (Gibco) and 1% penicillin (100 U/ml) and streptomycin (100 μg/ml; Beyotime, Haimen, China). The explants were kept stationary for 72 hrs in a humidified incubator at 37°C with a 5% CO_2_. Thereafter, culture media (DMEM supplemented with 10% (v/v) FBS and 1% penicillin and streptomycin) was refreshed twice/week. After the cells grew to confluence, they were trypsinized and passaged. Cells were used for experiments at passages 3–5. The purity of SMCs was confirmed by immunofluorescent staining for alpha smooth muscle actin (α‐SMA, 1:100; Millipore, Billerica, MA, USA), and the purity of the cells was around 90%. For α‐SMA staining, cells were washed with PBS and then fixed with 4% paraformaldehyde in PBS for 10 min. The fixed cells were permeabilizated with 0.2% Triton X‐100 in PBS and blocked for 1 hr in PBS containing 10% bovine serum albumin. They were then incubated with primary antibodies overnight at 4°C, followed by incubation with Alexa fluor 488‐conjugated donkey antimouse (Molecular Probes, Eugene, OR, USA) secondary antibody for 1 hr at room temperature and then mounted in mounting medium with DAPI.

### Immunohistochemistry

Sections of aortic wall embedded in paraffin were pre‐fixed in 4% paraformaldehyde. The 10‐μm thick transverse sections were deparaffinized and rehydrated through xylene and ethanol followed by fixation in 4% PFA for 10 min. The sections were then permeabilized with 0.5% Triton X‐100 and stained with anti‐α‐SMA (1:100; Millipore). The sections were counterstained with haematoxylin and eosin. All images were analysed with an Olympus BX41 microscope (Tokyo, Japan) equipped with a CCD camera and imaging software.

### Real‐time qPCR

Total RNA was extracted from aortic tissue and cells using Trizol reagent (Ambion, Foster City, CA, USA) according to the manufacturer's protocol. *miR‐145* expression was detected by RT‐qPCR using universal cDNA synthesis (Tiangen Biotech, Beijing, China) and a miRcute miRNA SYBR qPCR Detection Kit (Tiangen Biotech). U6 snRNA expression was used as an internal reference. The miR‐specific LNA PCR primer set and the primers for U6 were obtained from Exiqon. To detect *KLF5* and *MYOCD* mRNA expression, total RNA was harvested and reverse‐transcribed using the AccuPower RocketScript RT PreMix kit (Bioneer, Daejeon, Korea), and RT‐qPCR was performed using a 2× Greenstar qPCR Master Mix kit (Bioneer). β‐actin was used as an internal control.

### Western blotting

Protein from tissues and cells was extracted in RIPA buffer (Solarbio, Beijing, China). Protein concentrations were determined by the bicinchoninic acid method. Western blotting was used to detect protein expression. One hundred microgram of protein extract was separated by SDS‐PAGE (Beyotime) and transferred to PVDF membranes, which were blocked with 5% non‐fat milk for 1 hr. Membranes were incubated with primary antibodies overnight at 4°C. After washing three times in 1× Tris‐buffered saline with Tween‐20, membranes were incubated with secondary antibodies (Zsgb‐bio, Beijing, China) for 1 hr. Relative band intensities were evaluated using Quantity One (Bio‐Rad MP4000, Hercules, CA, USA). Primary antibodies were calponin and α‐SMA (1:500; Millipore), KLF5 (1:50; Abcam, Cambridge, MA, USA), MYOCD (1:200; Abcam) and β‐actin (1:500; Biolegend, San Diego, CA, USA).

### *miRNA* inhibitor transduction

A *miR‐145*‐specific inhibitor (5′‐AGGGATTCCTCCCAAAACTGGAC‐3′) and a *miR‐145*‐specific mimic (5′‐GTCCAGTTTTCCCAGGAATCCCT‐3′) were obtained from GenePharma (Suzhou, China). The inhibitor was transduced into cultured VSMCs using a red fluorescent protein (RFP)‐lentiviral vector at a final concentration of 5 × 10^6^ TU/ml according to the manufacturer's recommendations. The mimic was transduced into cultured VSMCs using a RFP‐lentiviral vector at a final concentration of 3 × 10^6^ TU/ml according to the manufacturer's recommendations. Briefly, the *miR‐145* inhibitor and a scrambled negative control (NC) construct were diluted in medium to a final concentration of 5 × 10^6^ TU/ml. The *miR‐145* mimic and a scrambled NC construct were diluted in medium to a final concentration of 3 × 10^6^ TU/ml, and cells were incubated with the constructs for 48 hrs. All transductions were performed in triplicate.

### Cell proliferation analysis

Cells from VL donors were seeded at 1 × 10^4^/well into 96‐well plates and transduced with *miR‐145* inhibitor or the NC construct. Cells from CR donors were seeded at 1 × 10^4^/well into 96‐well plates and transduced with *miR‐145* mimic or the NC construct. Forty‐eight hours after transduction, proliferation of these transduced cells was compared to those from CR and VL donors using the Cell Counting Kit‐8 (CCK‐8, Dojindo Molecular Technologies, Inc., Tokyo, Japan) according to the manufacturer's instructions. Absorbance was read at 450 nm using a microplate reader (Tecan‐Infinite M200 Pro, San Jose, CA, USA). Proliferation of VSMCs was also determined by a 5‐ethynyl‐2‐deoxyuridine (EdU) incorporation kit (RiboBio, Guangzhou, China) according to the manufacturer's instructions. The proliferation rate was calculated by normalizing the number of EdU^+^ cells in five random microscopic fields.

For cell cycle analysis, cells were harvested 48 hrs after *miR‐145* inhibitor or mimic transduction, washed twice with PBS, and fixed in 75% ethanol overnight. Cells were washed twice with PBS, incubated in 10 μl/ml RNaseA at 37°C for 30 min, and stained with 0.5 mg/ml propidium iodide (PI) at 4°C for 30 min. Finally, cells were washed and resuspended in 500 μl PBS, and DNA content was detected by flow cytometry (BD Diagnostics Mississauga, ON, Canada).

### Statistical analysis

All experiments were independently performed at least three times. Data are expressed as mean ± S.D. and analysed using two‐tailed unpaired Student's *t‐*tests for two groups or anova for three or more groups. *P* < 0.05 was considered statistically significant.

## Results

### *miR‐145* and α‐SMA expression in human aortas

Patients with CR and AD were diagnosed with atherosclerosis prior to surgery, while patients with CN and VL were not. Although the aortic samples from the four groups of patients were ‘normal’, we considered CR and AR donors the atherosclerotic group, while CN and VL donors were considered non‐atherosclerotic controls. The patients’ characteristics are summarized in Table 1. The sex of patients showed no significant differences between groups, but the non‐atherosclerotic patients were significantly younger (*P* < 0.05).

**Table 1:** Patient characteristics

	AR	CR	CN	VL	*p*‐value
Sex (M/F)	6/5	23/19	4/5	8/10	>0.05
Age (year)	50.7 ± 1.7	55.4 ± 1.1	43.7 ± 3.1	49.2 ± 1.9	<0.01
Hypertension	11	28	4	8	>0.01
Diabetes	5	18	2	8	>0.05

**Figure 1:**
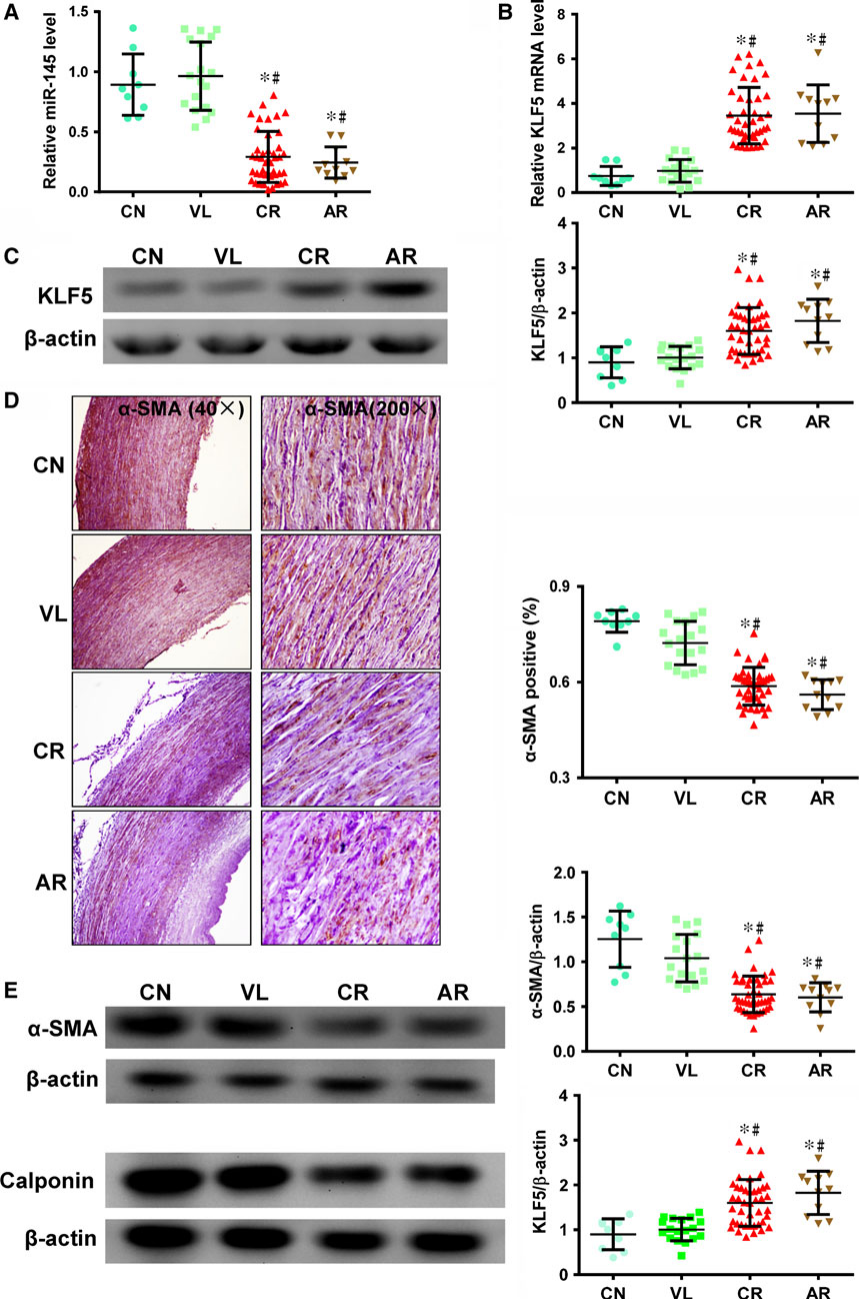
*miR‐145* expression and smooth muscle cell (SMC)‐specific contractile marker expression are reduced in aortas from patients with atherosclerosis‐associated cardiovascular disorders. (**A**) *miR‐145* expression was significantly higher in aortic samples from patients with non‐atherosclerosis‐associated congenital (CN) and valvular (VL) heart disease compared to tissue from patients with atherosclerosis‐associated coronary heart disease (CR) or aortic dissection disease (AR) by RT‐qPCR (*P < 0.05 *versus* CN, #*P* < 0.05 *versus* VL, AR:* n* = 11; CR:* n* = 42; CN:* n* = 9; VL:* n* = 18). (**B**) The mRNA (by RT‐qPCR) and (**C**) protein expression (by Western blot) of KLF5, a target of *miR‐145*, was significantly decreased in CN and VL donors compared with CR and AR donors (*P < 0.05 *versus* CN, #*P* < 0.05 *versus* VL). (**D**) Immunohistochemical staining for α‐SMA (brown) to identify SMCs. Quantification showed that tissue from CN and VL patients had a significantly higher percentage of α‐SMA ^+^ cells than CR and AR samples (*P < 0.05 *versus* CN, #*P* < 0.05 *versus* VL). (**E**) Expression of α‐SMA and calponin was significantly higher in CN and VL aortas than in CR and AR aortas (*P < 0.05 *versus* CN, #*P* < 0.05 *versus* VL). β‐actin was used as a loading control.

We found *miR‐145* expression to be significantly higher in CN and VL samples compared with CR and AR samples (Fig. 1A, *P* < 0.05 for both CN and VL patients *versus* both CR and AR patients), suggesting that *miR‐145* expression is decreased during atherosclerosis. Consistent with this, gene and protein expressions of KLF5, a *miR‐145* target, were significantly increased in atherosclerosis (Fig. 1B and C, *P* < 0.05 for both CN and VL patients *versus* both CR and AR patients for both protein and mRNA expression).

Since the *miR‐145*‐KLF5 pathway was shown to be critically required for VSMC contractile‐to‐proliferative phenotypic modulation 12, we assayed expression of SMC‐specific contractile markers. Immunohistochemistry against α‐SMA demonstrated that the contractile markers of VSMCs were significantly decreased in patients with atherosclerosis (Fig. 1D, *P* < 0.05 for both CN and VL patients *versus* both CR and AR patients). Western blots quantifying protein expression of contractile markers α‐SMA and calponin verified that patients with atherosclerosis‐associated disease had a decreased aortic contractile protein expression (Fig. 1E, *P* < 0.05 for both CN and VL patients *versus* both CR and AR patients for both proteins). These data suggest that *miR‐145* can be a modulator for contractile VSMC phenotypes in the human aorta, and its expression, along with that of SMC‐specific contractile markers, was decreased during atherosclerosis.

### VSMCs from patients with atherosclerosis showed decreased α‐SMA expression and increased proliferation

**Figure 2:**
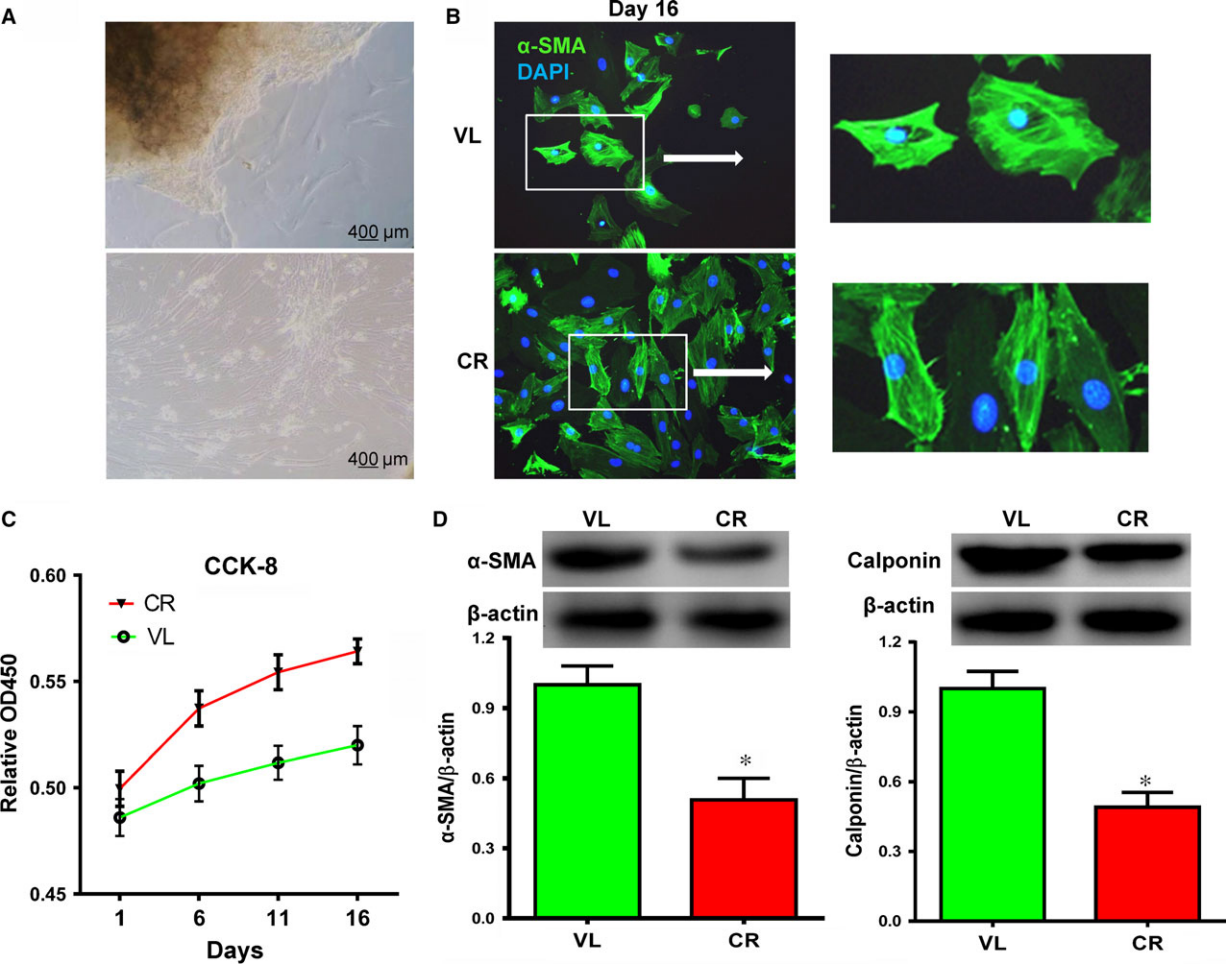
Aortic vascular smooth muscle cells (VSMCs) from patients with atherosclerosis‐associated coronary heart disease (CR) had lower SMC‐specific contractile marker expression and higher proliferation compared with patients with non‐atherosclerosis‐associated valvular disease (VL) *in vitro*. (**A**) VSMCs grown from patient aortic tissue show typical SMC morphology. (**B**) Immunofluorescent staining for α‐SMA (green) to determine SMC identity. Blue is 4′,6‐diamidino‐2‐phenylindole (DAPI)‐stained nuclei. (**C**) Cells from CR donors proliferate faster than cells from VL donors as determined by a CCK‐8 cell counting assay. (**D**) Western blotting showed that α‐SMA and calponin expression was significantly lower in cells from CR donors than from VL donors (*P < 0.05, *n* = 10). β‐actin was used as a loading control.

To exclude age as a confounding factor, we selected age‐matched samples from non‐atherosclerotic VL and atherosclerotic CR patients for VSMC culture. These patients’ characteristics are summarized in Table 2. We identified VSMCs morphologically and by α‐SMA staining (Fig. 2A and B). Proliferation of the cultured VSMCs from atherosclerotic CR donors was significantly higher than from non‐atherosclerotic VL donors (Fig. 2C). Additionally, we found that α‐SMA and calponin protein expression were significantly decreased in cells from CR donors compared with VL donors (Fig. 2D, *P* < 0.05 for both proteins). These results demonstrate that VSMCs from atherosclerotic CR patients showed increased proliferative and decreased contractile phenotypes compared with cells from non‐atherosclerotic VL patients.

**Table 2:** Patient characteristics of cell culture

	CR	VL	*p*‐value
Sex (M/F)	5/5	5/5	>0.05
Age (year)	54.9 ± 2.9	53.7 ± 2.8	>0.05
Hypertension	5	5	>0.05
Diabetes	6	5	>0.05

### VSMCs from patients with atherosclerosis showed decreased *miR‐145* expression

**Figure 3:**
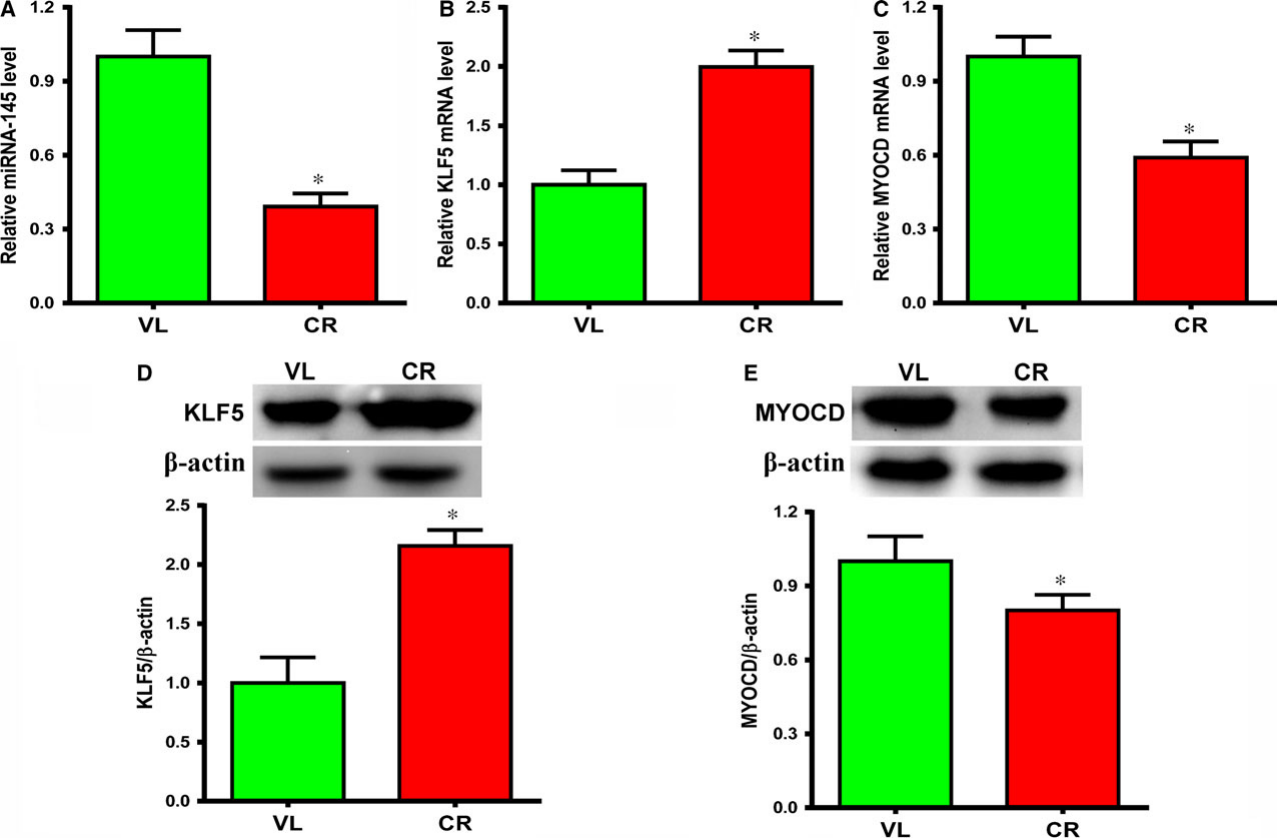
Aortic vascular smooth muscle cells (VSMCs) from patients with atherosclerosis‐associated coronary heart disease (CR) had lower *miR‐145* and MYOCD expression and higher KLF5 expression compared with patients with non‐atherosclerosis‐associated valvular disease (VL) *in vitro*. (**A** and **C**) *miR‐145* and MYOCD expression was significantly lower and (**B**) *KLF5* expression was significantly higher in cells from CR donors than in those from VL donors by RT‐qPCR (*P < 0.05, *n* = 10). (**D**) Western blotting showed protein expression of KLF5 was significantly higher (*P < 0.05, *n* = 10), and (**E**) protein expression of MYOCD was significantly lower in cells from CR donors than from VL donors (*P < 0.05, *n* = 4). β‐actin was used as a loading control.

To investigate a possible mechanism for the increased proliferation/decreased contractile phenotype of atherosclerotic VSMCs, we quantified *miR‐145* expression and function. We found that *miR‐145* expression was lower in VSMCs from atherosclerotic CR donors compared with non‐atherosclerotic VL donors (Fig. 3A, *P* < 0.05). Gene and protein expression of KLF5 and MYOCD was evaluated. MYOCD has a dominant role in the establishment and maintenance of a SMC contractile phenotype *in vivo* 18. Kruppel‐Like Factor 5, on the other hand, is a direct target of *miR‐145* with a protein sequence that is highly conserved across species. Previous work suggested that vascular injury causes downregulation of *miR‐145*, which leads to upregulation of KLF5 and inhibition of MYOCD 12. Consistent with previous findings, gene and protein expression of MYOCD were significantly lower in VSMCs from CR donors compared with VL donors (Fig. 3C and E, *P* < 0.05 for both gene and protein expression). This result is consistent with significantly increased KLF5 gene and protein expression in cells from CR donors relative to VL donors (Fig. 3B and D, *P* < 0.05 for both gene and protein expression). These results suggest that a more proliferative state of VSMCs from patients with atherosclerosis may be associated with lower expression of miR‐145.

### *miR‐145* regulated the expression of SMC‐specific contractile markers in VSMCs

**Figure 4:**
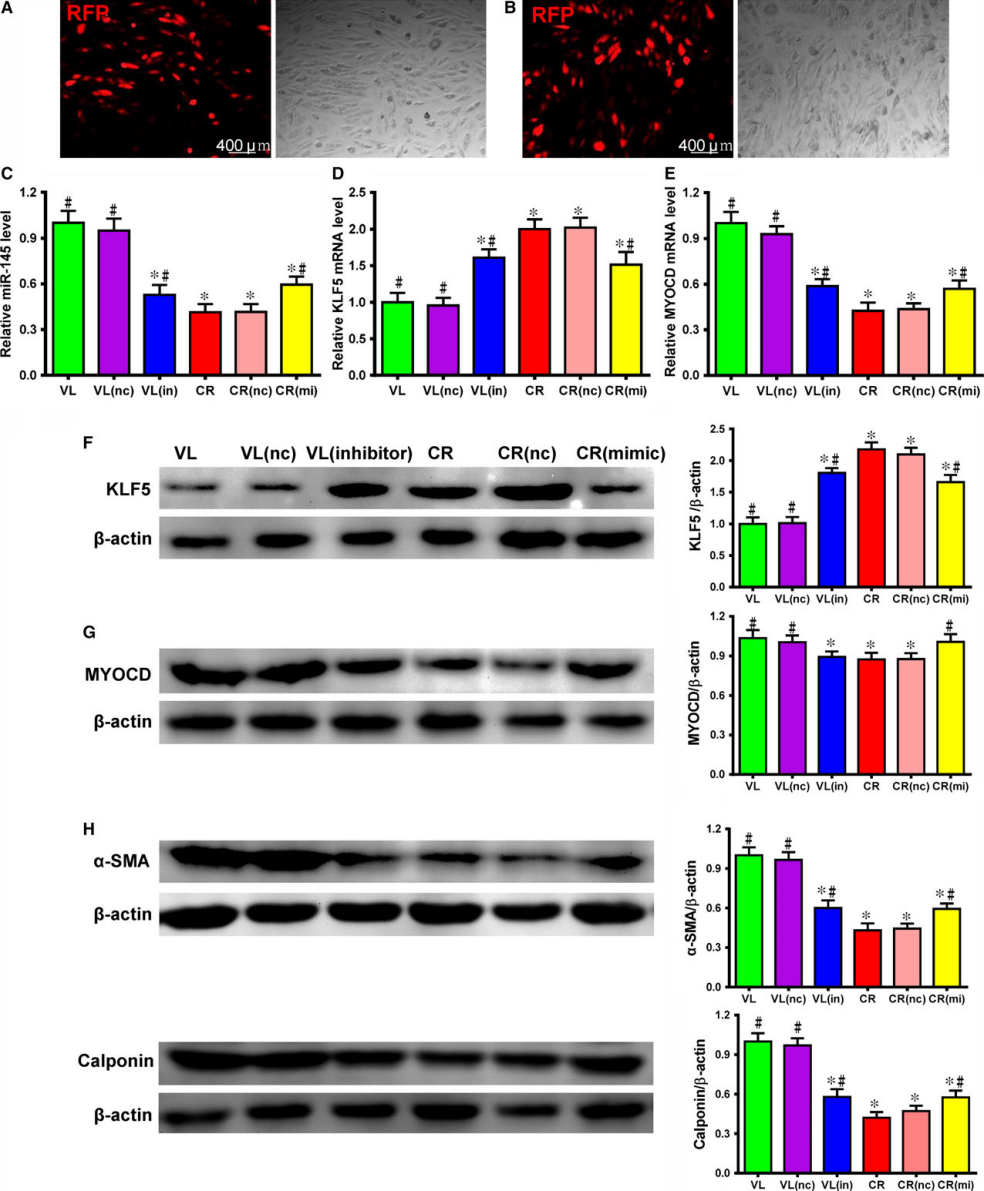
*miR‐145* inhibition in cells from patients without atherosclerosis decreased the expression of smooth muscle cell (SMC)‐specific contractile markers and its mimic in cells from patients with atherosclerosis increased the expression of smooth muscle cell (SMC)‐specific contractile markers *in vitro*. (**A**) A *miR‐145* inhibitor (in) was transduced into non‐atherosclerosis‐associated cells from valvular disease (VL) patients using a red fluorescent protein (RFP) tagged‐lentiviral vector. The RFP cotransduction marker demonstrated transduction efficiency. (**B**) A *miR‐145* mimic (mi) was transduced into atherosclerosis‐associated cells from coronary heart disease (CR) patients using a RFP tagged‐lentiviral vector. The RFP cotransduction marker demonstrated transduction efficiency. (**C**) After inhibitor transduction, *miR‐145* expression decreased in cells from VL donors and approached expression levels close to those found in non‐transduced cells from patients with atherosclerosis‐associated coronary heart disease (CR). After mimic transduction, *miR‐145* expression increased in cells from CR donors and approached expression levels close to those found in non‐transduced cells from patients with non‐atherosclerosis‐associated valvular disease (VL) by RT‐qPCR. VL/CR (nc) are cells transduced with a negative control scrambled inhibitor or mimic construct (*P < 0.05 *versus* VL, #*P* < 0.05 *versus* CR, n1 = 10, n2 = 4). After *miR‐145* inhibitor transduction, KLF5 (**D**) mRNA and (**F**) protein expression were increased in cells from VL donors and approached expression levels close to those found in cells from non‐transduced CR donors. After *miR‐145* mimic transduction, KLF5 (**D**) mRNA and (**F**) protein expression were decreased in cells from CR donors and approached expression levels close to those found in cells from non‐transduced VL donors as demonstrated by RT‐qPCR and Western blot (*P < 0.05 *versus* VL, #*P* < 0.05 *versus* CR, n1 = 10, n2 = 4). β‐actin was used as a loading control. After *miR‐145* inhibitor transduction, MYOCD (**E**) mRNA and (**G**) protein expressions were decreased in cells from VL donors and were similar to the expression levels of cells from non‐transduced CR donors. After *miR‐145* mimic transduction, MYOCD (**E**) mRNA and (**G**) protein expressions were increased in cells from CR donors and were similar to the expression levels of cells from non‐transduced VL donors as demonstrated by RT‐qPCR and Western blot (*P < 0.05 *versus* VL, #*P* < 0.05 *versus* CR,* n* = 4). β‐actin was used as a loading control. (**H**) After inhibitor transduction, α‐SMA and calponin protein expression decreased in cells from VL donors and approached expression levels close to those found in cells from non‐transduced CR donors. After mimic transduction, α‐SMA and calponin protein expression increased in cells from CR donors and approached expression levels close to those found in cells from non‐transduced VL donors as shown by Western blot (*P < 0.05 *versus* VL(nc), #*P* < 0.05 *versus* VL, n1 = 10, n2 = 4). β‐actin was used as a loading control.

We transduced *miR‐145* inhibitor into VSMCs from non‐atherosclerotic VL donors [VL(in), Fig. 4A] and *miR‐145* mimic into VSMCs from atherosclerotic CR donors [CR(mi), Fig. 4B]. After inhibitor transduction, *miRNA‐145* expression in VL cells significantly decreased compared with non‐transduced cells (VL) and cells transduced with a scrambled NC construct [VL(nc)]. The resulting *miRNA‐145* expression levels in VL[in] cells were close to those seen in cells from atherosclerotic CR donors. On the other hand, *miRNA‐145* mimic transduction in the CR cells [CR (mi)] significantly increased the expression of *miR‐145* though it did not completely restore it to the level of the VL group (Fig. 4C, *P* < 0.05). These results were corroborated by the significantly increased KLF5 gene and protein expression in the VL(in) group though it was still lower than that of the CR cells (Fig. 4D and F, *P* < 0.05). In contrast, KLF5 gene and protein expression was significantly decreased in the CR(mi) cells though it was still higher than that of the VL cells (Fig. 4D and F). Significantly decreased MYOCD gene and protein expression was found in the VL(in) cells which was comparable to that of the CR cells in responding to the increase in KLF 5 expression (Fig. 4E and G, *P* < 0.05). In contrast, significantly increased MYOCD gene and protein expression was seen in the CR(mi) cells which was comparable to the level of the VL cells (Fig. 4E and G). Next, we examined the contractile proteins such as α‐SMA and calponin. As expected, the protein expression of α‐SMA and calponin decreased in the VL(in) cells which were close to that of the CR cells (Fig. 4H, *P* < 0.05). On the other hand, the protein expression of α‐SMA and calponin was partially restored in the CR(mi) cells though it is still lower than that of the VL cells (Fig. 4H). These results suggest that *miR‐145* upregulates the expression of VSMC contractile markers and this might be achieved through its downstream targets, KLF5 and MYOCD.

### *miR‐145* inhibited VSMC proliferation

**Figure 5:**
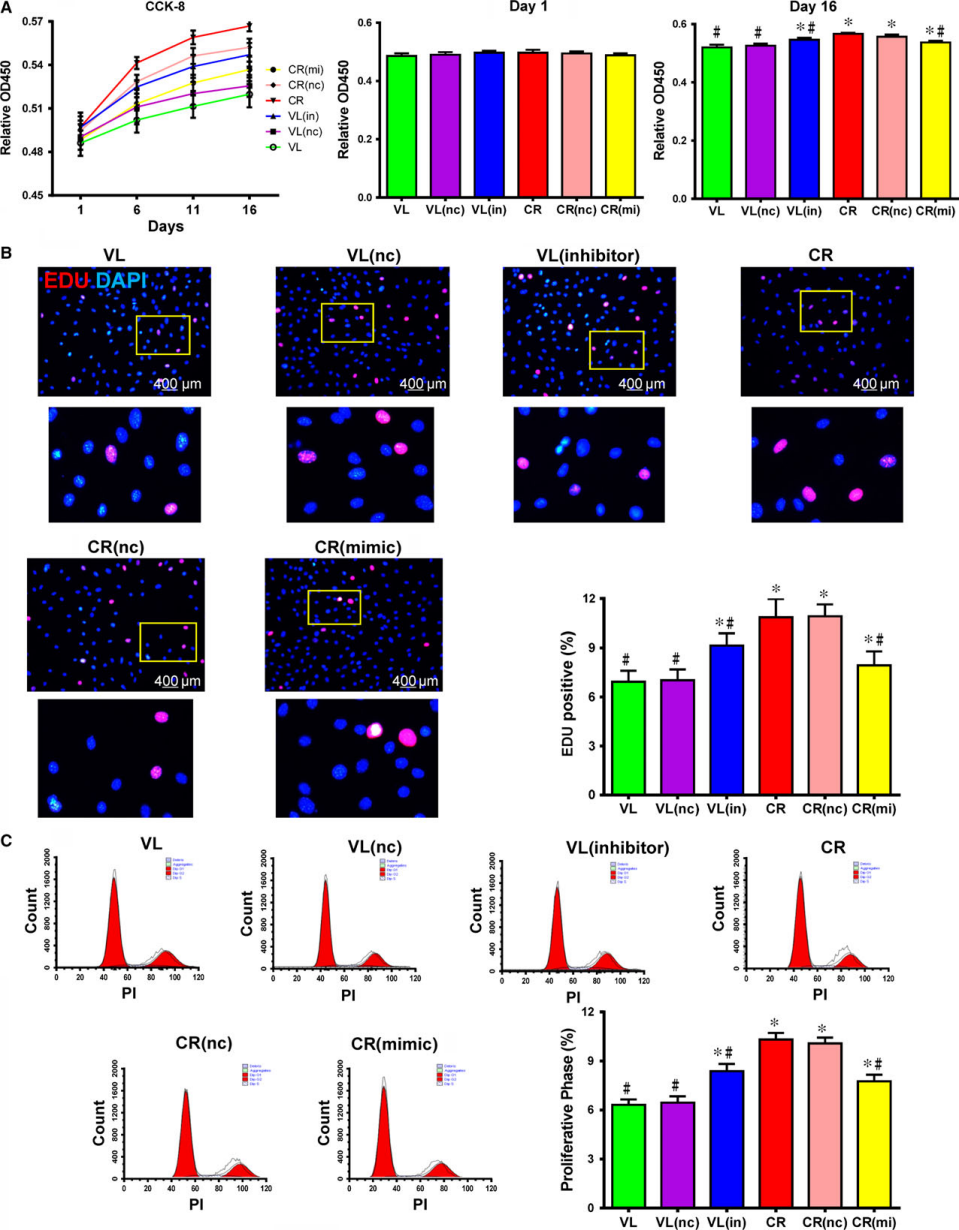
*miR‐145* inhibited human vascular smooth muscle cell (VSMC) proliferation *in vitro*. (**A**) Proliferation of *miR‐145* inhibitor‐transduced cells from non‐atherosclerosis‐associated valvular disease (VL) patients was similar to non‐transduced cells from patients with atherosclerosis‐associated coronary heart disease (CR), and proliferation of *miR‐145* mimic‐transduced cells from atherosclerosis‐associated coronary heart disease (CR) patients was similar to non‐transduced cells from patients with non‐atherosclerosis‐associated valvular disease (VL) as revealed by CCK‐8 cell counting 16 days after plating. VL/CR (nc) are cells transduced with a negative control scrambled inhibitor or mimic construct (*P < 0.05 *versus* VL, #*P* < 0.05 *versus* CR, n1 = 10, n2 = 4). (**B**) 5‐ethynyl‐2‐deoxyuridine (EdU) DNA synthesis marker (red) staining showed that the number of proliferating *miR‐145* inhibitor‐transduced cells from VL donors increased to a level similar to cells from non‐transduced CR donors and the number of proliferating *miR‐145* mimic‐transduced cells from CR donors increased to a level similar to cells from non‐transduced VL donors (*P < 0.05 *versus* VL, #*P* < 0.05 *versus* CR, n1 = 10, n2 = 4). (**C**) The number of *miR‐145* inhibitor‐transduced VSMCs from VL donors in a proliferative phase was similar to non‐transduced VSMCs from CR donors, and the number of *miR‐145* mimic‐transduced VSMCs from CR donors in a proliferative phase was similar to non‐transduced VSMCs from VL donors as shown by flow cytometry (*P < 0.05 *versus* VL, #*P* < 0.05 *versus* CR, n1 = 10, n2 = 4).

*miR‐145* may modulate the phenotype of VSMCs *via* KLF5 to promote the deleterious VSMC proliferative characteristic of atherosclerosis. To explore this idea, we evaluated VSMC proliferation using three assays: cell counting, DNA synthesis quantification and percentage of cells in proliferative phase by flow cytometric analysis for PI staining. The rate of VSMC proliferation (assessed by cell counting) and the percentage of VSMCs staining positive for DNA synthesis or in the proliferative phase were greater in the VL(in) cells than in the VL cells though they did not reach the levels of the CR cells. In contrast, the values of these three parameters were less in the CR(mi) cells though they were still higher than that of the VL cells (Fig. 5 A–C, *P* < 0.05 for all three assays). These data demonstrate that *miR‐145* inhibited VSMC proliferation *via* phenotypic switching through its target mediators, *KLF5* and MYOCD.

## Discussion

Increased detrimental cell proliferation plays a key role in proliferative vascular diseases such as atherosclerosis. While conventional atherosclerosis treatments may decrease risk factors such as circulating cholesterol and inflammation, their effect on VSMC proliferation is not clear 1. Genetic and metabolic interventions may, therefore, prove optimal for treating the detrimental VSMC proliferation that affects the development of atherosclerosis. Atherosclerotic lesions in patients with atherosclerosis are often in multiple locations, and research has focused on the development of atherosclerosis in the plaque area. In previous studies, plaque VSMCs were isolated from human endarterectomy specimens and subcultured *in vitro*, and their properties were compared with those of VSMCs derived from the media of normal arteries 19, 20, 21, 22. A striking characteristic of plaque VSMCs *in vitro* is their restricted capability to proliferate 19, 20, 21, 23, 24. Even though this feature is not universally found 25, isolated plaque‐derived VSMCs show lower rates of cell proliferation and a lower percentage of cells in the S phase of the cell cycle 21, 23. In addition, plaque VSMCs undergo premature senescence with high ‘senescence‐associated’ β‐galactosidase activity (SaβG) staining even at early stages of culture 26.

In this study, we investigated VSMC functional characteristics in ‘relatively normal aortic vessel walls’ from atherosclerotic and non‐atherosclerotic patients. Compared with VSMCs from atherosclerotic plaques, the VSMCs we isolated from human aortic walls (‘normal region’) from patients with atherosclerosis had the capability to proliferate and express proteins (α‐SMA, calponin) associated with the contractile phenotype. In addition, VSMCs derived from human aortic walls (‘normal region’) from patients with atherosclerosis had a higher percentage of cells in the S phase of the cell cycle (~10%) compared with VSMCs from atherosclerotic plaques (~3.5 ± 1.2%). Conversely, we found that VSMCs from atherosclerotic patients had greater proliferative capacity compared with VSMCs from the same area in non‐atherosclerotic patients which was accompanied by lower expression of contractile proteins (α‐SMA, calponin) and that *miR‐145* tightly regulates this proliferative capacity. Atherosclerosis can be considered to be a form of chronic inflammation resulting from interaction between modified lipoproteins, monocyte‐derived macrophages, T cells and the normal cellular elements of the arterial wall. This inflammatory process can ultimately lead to the development of complex lesions or plaques. Therefore, the ‘relatively normal aortic vessel walls’ adjacent to the plaques are also under attack by chronic inflammation and may undergo phenotypic switch. These data provide important information for the prevention of atherosclerotic development in patients with atherosclerosis.

Unlike some differentiated cells, VSMCs retain plasticity and can modulate their phenotype in response to changes in the local environment. In response to vascular injury, VSMCs can switch from a contractile to a proliferative state 27, 28. Several studies have demonstrated that deregulation of VSMC function plays a critical role in the pathogenesis of many proliferative vascular diseases, including hypertension and atherosclerosis 4. For example, mechanical stretch due to increased blood pressure or hemodynamic forces, such as shear stress, can modulate VSMC phenotypic changes 4, 29, 30, 31.

Recently, *miR‐145* has been reported to suppress cell proliferation, invasion and migration in some carcinomas 32, 33, 34, 35, 36, 37, and it can function as a biomarker for their diagnosis 38, 39, 40. The anti‐proliferative effect of *miR‐145* on VSMCs appears to be mediated by restoration of a contractile phenotype. It has been shown that restoring *miR‐145* expression limited neointima formation in response to vascular injury by promoting KLF5 downregulation and VSMC contractile protein expression 12, 15, 41. Previous studies have also demonstrated an important role for *miR‐145* in VSMC differentiation and function by direct and indirect modulation of MYOCD 16 and actin polymerization 42. Moreover, *miR‐145* knockout mice show lower systolic and diastolic blood pressure 7, mainly due to reduced contractile tone in small‐resistance arteries. Indeed, it has been reported that *miR‐145* is required for stretch‐induced VSMC differentiation 15 and contractile phenotype acquisition 7. However, detailed molecular mechanisms for this phenotypic modulation in human vessels, especially at a ‘relatively normal aortic region’ of the blood vessel of patients with atherosclerosis, have not been studied.

In this study, we demonstrated a significant difference in *miR145* and MYOCD levels in samples from atherosclerotic and non‐atherosclerotic patients. However, the interaction between *miR‐145* and MYOCD was not investigated, but could be ‘feed‐forward reinforcement’ as described by Cordes *et al*. 16. They showed that *miR‐145* and *miR‐143* were direct transcriptional targets of serum response factor (SRF), MYOCD and Nkx2.5. Serum response factor weakly activated the *miR‐143/145* enhancer upstream of a luciferase reporter, but co‐transfection of MYOCD synergistically and robustly activated luciferase activity in Cos cells. On the other hand, *miR‐145* increased luciferase activity in the cells transfected with the CMV‐luciferase‐MYOCD 3′ UTR reporter. These findings suggest that miRNAs can act as translational activators or repressors depending on the state of the cell cycle 43. *miR‐145* may promote VSMC differentiation in part by increasing MYOCD protein and functioning in a feed‐forward reinforcement of its own expression by the SRF–MYOCD complex. However, the functionality of the *miR‐145* site in the *MYOCD* gene was not directly addressed in the current study. Cordes *et al*. clearly demonstrated that the *miR‐145* site in MYOCD 3′ UTR is dispensable for the feed‐forward loop which is likely due to the effects on SRF and the delicate balance of SRF interactions with cofactors exerting positive and inhibitory effects on transcription 16. Wang *et al*. showed that growth signals repress smooth muscle genes by triggering the displacement of MYOCD from SRF by Elk‐1, a ternary complex factor (TCF) of the ETS‐domain family that acts as a myogenic repressor 44. MYOCD and Elk‐1 compete for a common docking site on SRF to exert opposing influences on smooth muscle gene expression 44. Indeed, as previously reported, SRF activates genes involved in smooth muscle differentiation and proliferation by recruiting muscle‐restricted cofactors, such as the transcriptional coactivator MYOCD and TCFs of the ETS‐domain family, respectively 45, 46. Kruppel‐Like Factor 5, on the other hand, contains a binding site for *miR‐145* with a protein sequence that is highly conserved across humans, mice and rats. Vascular injury causes downregulation of *miR‐145* which leads to upregulation of KLF5 and inhibition of MYOCD. Therefore, rather than directly acting on a *miR‐145* binding site in the 3′ UTR of MYOCD, the release of *miR‐145* repression on the negative MYOCD regulators, KLF4 and/or KLF5 may be the main reason for the subsequent attenuated expression of MYOCD 12.

The role of *miR‐145* in human patients with various pathological conditions has been examined. Upregulation of *miR‐145* was observed in the lung tissue of patients with idiopathic and heritable PAH compared with unaffected control subjects 14. Elevated expression levels of *miR‐143/5* in saphenous vein SMCs from patients with Type 2 diabetes were found to drive persistent changes in phenotype and function 47. The role of *miR‐145* in cardiovascular pathophysiology and atherosclerosis development in humans has also been explored 17. There are profound differences in the expression of 5 miRNAs including *miR‐145* (*miRNA‐100, miRNA‐127, miRNA‐133a and miRNA‐133b*) in symptomatic *versus* asymptomatic plaques. In a subsequent study, the same group found that *miR‐145* was significantly more expressed in atherosclerotic plaques of hypertensive patients than in control plaques 2. *miR‐145* is also modulated in carotid atherosclerotic plaques after ischaemic stroke 17, suggesting that it may play a role in the development of atherosclerosis.

In this study, we explored the association between *miR‐145* and aortic atherosclerosis and found that *miR‐145* was significantly reduced in human aortic walls and cultured VSMCs from patients with vascular atherosclerosis. We directly compared the expression levels of *miR‐145* along with the downstream mediators/contractile proteins in the non‐atherosclerotic aortic vessel walls from patients with and without vascular atherosclerosis, which has not been reported previously. Furthermore, the effects of *miR‐145* inhibition or promotion on VSMC phenotype switch were examined using the VSMCs from aortic vessel walls of patients with and without atherosclerosis.

We found that *miR‐145* expression was decreased, while expression of transcription factor KLF5, a known *miR‐145* target, was concomitantly increased in atherosclerotic aortas relative to non‐atherosclerotic aortas. This suggests that *miR‐145* may modulate the phenotypic switch of VSMCs from a contractile to a proliferative state *via* KLF5. In fact, we found lower SMC‐specific contractile marker expression (calponin and α‐SMA) in the aortas from patients with atherosclerosis compared with those from patients without atherosclerosis. These findings suggest that *miR‐145* may control the adverse switch of VSMCs from a contractile to a proliferative phenotype during atherosclerosis. Indeed, we found that cultured VSMCs from patients with atherosclerosis had decreased expression of the contractile markers calponin and α‐SMA, along with decreased expression of *miR‐145* and MYOCD and increased expression in KLF5 compared with the VSMCs from patients without atherosclerosis. *miR‐145* mimic‐transduced VSMCs from patients with atherosclerosis showed increased expression of calponin and α‐SMA and decreased proliferation compared with non‐transduced controls, and these expression and proliferation levels were close to the levels found in cells from patients without atherosclerosis. Conversely, *miR‐145* inhibitor‐transduced VSMCs from patients without atherosclerosis showed decreased expression of calponin and α‐SMA and increased proliferation compared with non‐transduced controls, and these expression and proliferation levels were close to the levels found in cells from patients with atherosclerosis‐associated cardiovascular disease.

Our data demonstrate that *miR‐145* modulates the phenotypic switch of VSMCs from a contractile to a proliferative state *via* KLF5 and MYOCD and show that *miR‐145* inhibits human VSMC proliferation in cells from atherosclerotic patients. These findings suggest that *miR‐145* controls the adverse phenotypic switch of VSMCs during atherosclerosis. Given this role and the role of *miR‐145* in adverse remodelling during other cardiovascular diseases 14, this particular *miRNA* may represent an important future therapeutic target.

### Study limitations

In this study, we used cultured cells from non‐defected areas of the aorta in patients with atherosclerosis to indirectly show that *miR‐145* modulates the phenotypic switch of VSMCs from a contractile to a proliferative state *via* KLF5 and MYOCD in atherosclerosis. However, there is a possibility that an epigenetic memory may get uncovered upon plating the cells (which is not physiological) or it may not be relevant in the context of the organ itself. In addition, critical controls such as VSMCs from the atherosclerotic plaques are lacking in the present study. Future studies with freshly isolated tissue from non‐defected areas of the aorta and from atherosclerotic plaques are required to confirm the current findings in physiological conditions.

## Conflicts of interest

The authors confirm that there are no conflicts of interest.
